# What have molecular simulations contributed to understanding of Gram-negative bacterial cell envelopes?

**DOI:** 10.1099/mic.0.001165

**Published:** 2022-03-16

**Authors:** Syma Khalid, Cyril Schroeder, Peter J. Bond, Anna L. Duncan

**Affiliations:** ^1^​ Department of Biochemistry, University of Oxford, South Parks Road, Oxford, OX1 3QU, UK; ^2^​ Bioinformatics Institute (A*STAR), Singapore 138671, Singapore; ^3^​ Department of Biological Sciences, National University of Singapore, Singapore 117543, Singapore

**Keywords:** molecular dynamics, bacterial cell envelope, membrane protein, periplasm, molecular simulation

## Abstract

Bacterial cell envelopes are compositionally complex and crowded and while highly dynamic in some areas, their molecular motion is very limited, to the point of being almost static in others. Therefore, it is no real surprise that studying them at high resolution across a range of temporal and spatial scales requires a number of different techniques. Details at atomistic to molecular scales for up to tens of microseconds are now within range for molecular dynamics simulations. Here we review how such simulations have contributed to our current understanding of the cell envelopes of Gram-negative bacteria.

## Introduction

Bacterial cell envelopes are complex, multi-compartment molecular architectures that provide the relatively simple cells they surround with a sophisticated protection and surveillance system. They have been a source of fascination and focus of experimental study for hundreds of years – for example, the Gram-stain was developed in 1884. In the ensuing years a great amount of progress has been made in terms of identification, structural characterization and functional analyses of individual components of bacterial cell envelopes. But there are still a number of fundamental unknowns, which range from the precise function of individual proteins/protein complexes, to the organization of membrane proteins with respect to the lipids in the membranes, and the morphology of the cell wall. In many cases, the links between molecular structures and their cellular functions are still unclear. Often, but not always, this is due to a lack of understanding of the dynamics of those molecules. Classical molecular dynamics (MD) simulations provide a physics-based framework to accurately describe the motions of atoms within molecular systems. In recent years, advancements in models and methodologies, coupled with the availability of increasing computational power have resulted in MD simulations becoming an established technique, often employed in tandem with experimental methods, for the study of bacterial cell envelope structure and function [1, 2].

Here we provide a broad review of insights into the structure-dynamics relationships of bacterial cell envelopes derived from MD simulations. We discuss insights into (i) mechanistic aspects of specific proteins/protein assemblies, in the inner membrane (IM), outer membrane (OM) and within the periplasm, (ii) physical/mechanical properties of the cell envelope, and (iii) organization of the various components of the cell envelope, finishing with an outlook for the future. We mostly focus on Gram-negative bacteria as this reflects a large part of the simulation literature on bacterial cell envelopes. Given space constraints, we have restricted this review to a detailed look at a handful of examples from each component of the cell envelope with an emphasis on the biological implications of the results without delving too deeply into the details of the computational methodologies employed. Historically, notwithstanding studies of simple phospholipid bilayer models, investigations of individual bacterial proteins were reported before those probing the higher organisation of the cell envelope. However, here we have taken the approach of discussing the bigger picture first, then ‘zooming in’ on the details of individual proteins/proteins complexes.

## Organisation of the cell envelope

Structural biology has provided a wealth of information regarding individual bacterial cell envelope proteins and protein complexes/assemblies, with cryogenic electron microscopy (cryo-EM) in particular yielding increasingly larger structures at medium to high resolution in recent years. However, the localization of macromolecular components within the tripartite cell envelope, the details of inter-protein dynamics, and the impact of the local physiological environment are still difficult to address with a single technique in isolation. Here, we discuss some examples in which MD simulations have provided information in these areas. Large-scale models and simulations of bacterial membranes, and more recently both membranes and the periplasm, have been used to investigate the effects of complex lipid compositions and protein crowding on the organisation of bacterial envelopes, often making use of coarse-grained (CG) force fields. In traditional MD simulations, all heavy atoms and either all or only polar hydrogen atoms are explicitly included in the system – CG simulations simplify such representations, effectively treating them as lower resolution versions, thereby enabling access to longer time and/or length scales. In the most popular CG models ~four heavy atoms are combined into single atom-like particles [3–5]. In the following examples, both CG and traditional methods have been employed.

While it is known from a number of experimental and computational studies that the slow diffusion of bacterial lipopolysaccharide (LPS) as well as the tight crosslinking of the LPS phosphate groups by divalent cations render the OM highly impermeable, simulations have gone one step further in quantifying the differences in barriers to permeation of a number of organic molecules across the two leaflets of the OM, thanks to the capacity to calculate the associated free energies [6]. The simulation data show distinctly asymmetric barriers for permeation across the two leaflets of the OM. The lipid A headgroups of LPS provide a bigger barrier to permeation of hydrophobic molecules compared to the headgroup regions of the phospholipids of the inner leaflet, while the low dielectric acyl core of the membrane is almost equally unfavourable in both leaflets for polar molecules. This study also demonstrated the slower diffusion of molecules in the outer leaflet compared to the inner leaflet of the OM. Thus, given this slow movement of LPS and the energetic barriers to permeation, the key questions that arise regarding the OM are how are: (i) molecular permeation across the OM, and (ii) positioning of OMPs achieved?

**Fig. 1.**
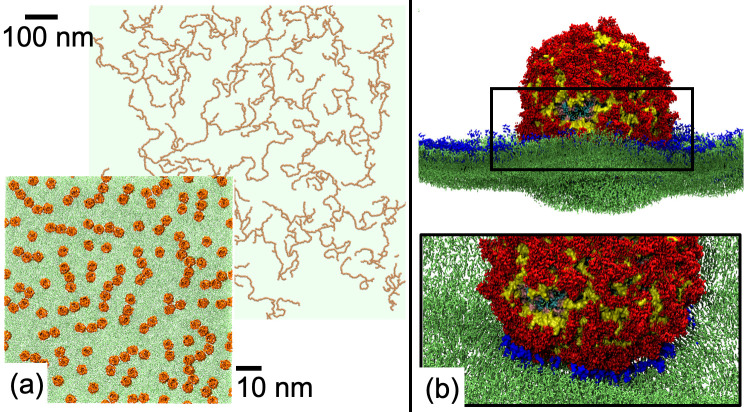
(a) Models of BtuB (orange) in lipid bilayers (green) at two different levels of coarse-graining [8]. (b) Interaction of an OMV containing O-antigen level LPS (red and yellow) with a host plasma membrane composed of a mixture of lipids (green) with gangliosides highlighted (blue) [22].

In perhaps the earliest large-scale simulation study of a realistic OM model, Holdbrook *et al*. reported CG simulations of spherical vesicles to understand the interplay between OMPs and lipids [7]. Microsecond-timescale simulations were performed of vesicles with radii of up to 32 nm, containing up to 64 copies of the trimeric porin OmpF embedded within lipid bilayers composed of either a mixture of phosphatidyl-ethanolamine (PE) and phosphatidyl-glycerol (PG), or di-stearoyl-phosphatidyl-choline (DSPC) and di-lauroyl-phosphatidyl-choline (DLPC) lipids. Lipid composition affected both local and global vesicle undulations, which it was postulated, may drive OmpF assembly. Protein and lipid diffusion were correlated over distances reaching up to 6 nm from the protein surface, which is suggestive of a mechanism for long-range communication in protein sorting and aggregation. In a subsequent study of OM organization, Chavent *et al*. reported large-scale CG MD simulations of the OMPs BtuB and OmpF, alongside mesoscale simulations [8]. CG MD simulations of 144 BtuB or 100 OmpF trimers in a bilayer composed of PE and PG lipids were run for microseconds. Additionally, much larger CG MD simulations were performed, containing 2304 protomers lasting 2.5 µs. These simulations directly mimicked length scales from fluorescence microscopy and demonstrated heterogeneity in protein cluster formation. However, due to the computational challenge of analysing such a large system, it was apparent that a less computationally expensive model was required, which led to the development of a mesoscale model. The CG MD simulations identified interaction interfaces of BtuB and OmpF, which were used to parametrize the mesoscale models. Mesoscale simulations of 2500 BtuB molecules were performed over millisecond timescales and allowed direct comparison with fluorescence microscopy data (Fig. 1). The mesoscale simulations predicted how protein interaction interfaces underpin the formation of OMP islands [9]. Notably, such OMP island have recently been directly imaged with atomic force microscopy by Hoogenboom and co-workers [10].

Pandit and Klauda described a model of the *E. coli* inner membrane, which included the hitherto largely neglected lipids containing a cyclopropane ring within their acyl chains [11]. It was found that the inclusion of the cyclopropane ring yielded more rigid and thinner membranes compared to those containing only mixtures of POPE and POPG, with thinner membranes better matching the width of the hydrophobic patches on the surfaces of *E. coli* transmembrane proteins. Large-scale simulations of the inner membrane are less prevalent in the literature, however a noteworthy example is the all-atom study of Sener *et al*. [12]. A combination of cryo-EM, nuclear magnetic resonance (NMR), x-ray crystallography and atomic force microscopy (AFM) data was used to construct a model of the photosynthetic unit (PSU) of purple photosynthetic bacteria, which consists of light-harvesting complexes and reaction centres, and measures ~70 nm in diameter. The construction of such a model allowed for simulations investigating short (50 ps) excitation lifetimes and high quantum efficiency of the PSU and led to a greater understanding of excitation dynamics within tightly packed arrays.

A comparative CG simulation study of protein and lipid dynamics and their correlation was reported by Hsu *et al*. [13]. It was shown that protein/LPS motion is highly coupled over large distances, whereas protein/phospholipid motion is only transiently coupled, and over shorter distances. This study highlighted for perhaps the first time that OMPs experience/induce very different dynamics within the two leaflets of the OM. Furthermore, the relationship between membrane stiffness and curvature and the surface area of the membrane occupied by proteins was also explored. In this study, the location of the two membranes was determined by using the full-length AcrAB-TolC RND pump as a guide, but the cell wall was omitted due to the lack of a CG model and paucity of details regarding the physiological location of the cell wall with respect to the protein. We note here that CG models of the *E. coli* cell wall have now been reported [14, 15].

Determining the location of the protein with respect to the cell wall within the periplasm is a problem often encountered when setting up large multicomponent simulations, given that portions of the cell wall are rarely present in protein structures. This is particularly true when simulating protein complexes that span the cell envelope. It is then more instructive to turn to electron cryotomography (cryo-ET) data for such information. This was the approach taken by Gumbart and co-workers when investigating the *E. coli* AcrAB-TolC efflux pump, in which simulations initiated from a cryo-EM structure were employed to determine the location of the cell wall [16]. Comparison of MD and cryo-ET data showed excellent agreement. Similarly, the architecture of glycan strands in the Gram-positive bacterium *Bacillus subtilis* was investigated using cryo-ET and MD simulations, showing that they run circumferentially around the cell, which was already known to be the case in Gram-negative bacteria [17]. The experimental data was only reproduced in the atomistic simulations with the circumferential topology, which contradicts two alternative models in which the glycan strands are either perpendicular to the cell surface or present as coiled cables. Such approaches have also been used for inner membrane complexes. For example, Burt *et al*. used cryo-ET and subtomogram averaging to determine a three-dimensional map of a complete core signalling unit, a building block of bacterial supramolecular signalling arrays needed for stimulus detection and transmission [18]. Combining this map with previously resolved high-resolution structures and MD simulations enabled construction of a spatial model of the transmembrane core signalling units with accurate localisation of the individual domains.

## Mechanical strength of the cell envelope

In addition to the localization and dynamics of the individual components, MD simulations have provided details of their physical properties and contributions to the mechanical strength of the cell envelope. If we consider atomistic level physical characterization, insights from simulations have perhaps been most significant for the OM. Early papers in which force-field parameters for LPS were reported also provided atomistic details of cation-binding modes and slow diffusion rates of the LPS molecules.

Studies of the mechanical strength of cell-envelope components include work by Hwang *et al*. and Jefferies *et al*. [19, 20]. The former investigated how the cell wall, IM and OM collectively protect the cell from osmotic pressure – which arises when the cytoplasm and external environment have different solute concentrations – by distributing it among them [19]. To determine how the structural characteristics of these structures correlate with the mechanical resistance of the cell and how the envelope components respond to changes in surface tension, atomistic MD simulations of the individual components were compared with experimental data on monolayers representative of the simulated systems. This yielded mixed agreements for the area compressibility *K*_A_, which was rationalized by the slow diffusion of LPS and limited simulation timescales, making LPS leaflets adopt crystalline properties, as well as other experimental factors such as the presence of more compressible disordered regions also possibly playing a role. Furthermore, the study unveiled that expansion makes membranes become softer while the cell wall stiffens at mid to high tensions. These findings were combined in a model where the OM and cell wall share the osmotic pressure below 0.3 atmosphere (atm) and the cell wall carries the majority of the tension at pressures above 1 atm. Jefferies *et al*. employed MD simulations on OM models containing smooth (incorporating an O-antigen chain) and rough (without O-antigen) LPS in the outer leaflet and phospholipids in the inner leaflet [20]. This work showed the effect of the lipid environment of smooth LPS lipids on the orientation and clustering of their O-antigen chains: when only smooth LPS was present, the O-antigen chains were packed tightly, which resulted in strong cohesive intermolecular interactions, as opposed to simulations where interstitial phospholipids and rough LPS lipids were added, resulting in tilted O-antigen chains with lower cohesion. The group thus established that in Gram-negative membrane models, lipid mobility and membrane mechanical strength are affected by the packing of the O-antigen chains, with membranes composed of only smooth LPS being able to withstand higher surface tensions than those additionally containing rough LPS lipids.

Outer membrane vesicles (OMVs) are nano-sized proteoliposomes secreted from the bacterial cell envelope. OMVs contain and deliver a wide range of cargos including toxins and virulence factors to host cells, which gives them a significant role in pathogenesis. Studying the process of host cell entry using experimental methods is difficult, particularly at molecular level [21]. The interaction of OMVs with host cells and in particular the impact of the mechanical strength imparted by the O-antigen chain on OMVs was investigated by Jefferies and Khalid via large scale CG simulations [22]. In particular it was asked why OMVs comprised of O-antigen are more readily incorporated into host cells compared to those containing shorter LPS molecules [21]. OMVs of radius 20 nm, which contained a mixture of lipids (to reflect *E. coli* outer membranes), were simulated together with two membrane types, a simple phospholipid bilayer and a patch of a complex (generalized) plasma membrane model previously reported by Sansom and co-workers [23]. The OMVs included either smooth or rough LPS in their outer leaflets. As expected from the earlier study of mechanical strength, the rough OMVs were more deformable. Crucially, the smooth OMVs did not induce curvature of the phospholipid bilayers, nor did they themselves deform, however when interacting with the model plasma membrane, they organized domain-forming lipids creating a ‘landing platform’ after which the plasma membrane showed signs of curvature (Fig. 1). This type of mechanism is known to be exploited by some viruses [24]. In summary, MD simulations have provided insights into the respective roles of the membranes and cell wall in responding to external pressure such as surface tension, and focussing on the outer membrane, they have shown the effects of differing LPS lengths on not only mechanical strength of the bacterial membrane, but also the ability to induce lipid ordering in host membranes.

We now turn our attention to insights gained from MD simulations into the behaviour and function of proteins that are native to the two membranes and the periplasm of the cell envelopes of Gram-negative bacteria. As with the earlier sections, there are far more studies in the literature than can be described here, so we have chosen instructive examples of proteins from each of the three cell envelope compartments with explanations for the choices.

## Outer membrane proteins

### Conformational dynamics in simple membrane models

Native OMPs have been simulated since the early 2000s when they were first modelled in simple phospholipid bilayers that mimicked many of the *in vitro* experimental setups [25, 26]. An early success story for MD simulations was the study of the small *E. coli* protein OmpA by Bond, Sansom and co-workers [27]. OmpA is a multidomain protein; the N-terminal domain is an eight-stranded barrel connected via an unstructured linker region to the soluble C-terminal domain, which can bind to the cell wall. The available x-ray structures of the barrel domain at the time suggested the absence of a continuous pore within the barrel. This was puzzling given that a number of groups had reported channel conductance across OmpA. MD simulations of the barrel domain of OmpA in a 1,2-dimyristoyl-*sn*-glycero-3-phosphocholine (DMPC) lipid bilayer showed that a small perturbation of a salt bridge formed between arginine and glutamate side chains within the centre of the barrel was sufficient to enable complete permeation of water molecules through the protein. Subsequent NMR and mutational studies showed that a perturbation of the salt bridge in this region does indeed lead to an open pore [28]. The same group studied the conformational dynamics of a number of different OMPs from both a fundamental biology and biotechnology perspective [29–32]. Importantly, they studied dynamics of a number of OMPs within flat phospholipid bilayers compared to within detergent micelles, which revealed more dynamic protein structures in the latter environment and highlighted the utility of MD simulations providing a route to bridging x-ray and NMR data [33, 34].

The trimeric general porins of *E. coli*, OmpF and OmpC, have been the focus of numerous simulation studies, which have yielded insights, for example, into the anion- and cation-binding sites within OmpF and the possible origins of its cation selectivity [35, 36]. Comparative studies of the two porins have been used to characterize and contrast their conformational dynamics as a function of temperature [37, 38]. Much of the simulation work on these general porins as well as more recent work on the specific OM channels of, e.g. *P. aeruginosa* has focussed on the recognition, binding and subsequent permeation of antibiotics through these proteins. Often, such studies are comprised of both experimental and computational contributions, each providing information complementing the other. In general, the simulations have been able to provide details of antibiotic-protein interactions, hydration levels of protein channels, flexibility of internal protein cavities as well as other conformational details that are not available from static structures. Due to space limitations we are unable to describe these studies in detail here, but we refer the reader to a number of key recent papers and a review [39–42].

### Outer membrane proteins and lipopolysaccharide

Atomistic models of LPS now exist for the AMBER, GROMOS and CHARMM families of force fields, enabling simulations of more biologically representative models of the OM [1, 43–46]. These simulations have the caveat that the slow-moving nature of LPS necessitates much longer simulation times to achieve equilibrated systems and to observe significant dynamics [47, 48]. This can in part be mitigated by the use of CG models. As a reminder, in CG simulations, the atomistic resolution is sacrificed for the ability to run longer simulations and study larger systems [49]. Nevertheless, atomistic simulations have been able to highlight the impact of LPS on, for example, the extracellular loops of OMPs. A study of the interactions between the OMP OprH from *P. aeruginosa* with LPS in OM models of both *P. aeruginosa* and *E. coli* revealed that the chemical composition of LPS molecules influences the structure and dynamics of the OprH loops, which in turn affects the stability of the OM [50].

There have been a number of simulation studies of TonB dependent transporters in model OMs. Such simulations have shown that the large extracellular loops of OMPs exhibit different, not just slower, dynamics in bilayers with LPS in the outer leaflet compared to those with only phospholipids in both leaflets, as seen for example in comparative simulations of the TonB-dependent transporter FecA in LPS-containing membrane models and simple phospholipid bilayers [51]. Another TonB-dependent transporter, BtuB, is responsible for transporting Vitamin B12 into the cell. Atomistic simulations of BtuB showed that the oligosaccharides of LPS as well as Ca^2+^ ions stabilize the extracellular loops [52]. The extracellular loops were also shown to play a key role in the transport of Vitamin B12, whereby the mobility of the loops enables correct orientation of Vitamin B12 [53]. The importance of LPS and Ca^2+^ interactions with OMPs has been shown from simulations of OMPs other than TonB-dependent transporters. For example, key LPS-loop interactions were identified in a study of the permeation of arginine through OprD from *P. aeruginosa* [54]. Here the data suggested that the interaction between LPS and arginine plays a role in capturing the latter from the external medium and promoting its binding to the long extracellular loops of OprD prior to entering the protein for passive transport across the OM. The role of Ca^2+^ in stabilizing the protein was also observed in simulations of the *Enterobacter cloacae* protein OmpE36, which showed that calcium ions also play a role in mediating interactions between LPS and the protein; this may well be a more general phenomenon that applies to other OMPs [55]. Thus, while it is generally accepted that divalent cations cross-link the phosphorylated moieties of LPS, which renders the outer leaflet of the OM highly impermeable, there is increasing evidence from MD simulations that interactions between divalent cations, LPS and extracellular loops of OMPs are highly complex and likely cross-stabilize each other as well as facilitating orientation/entry of substrates into OMPs.

**Fig. 2.**
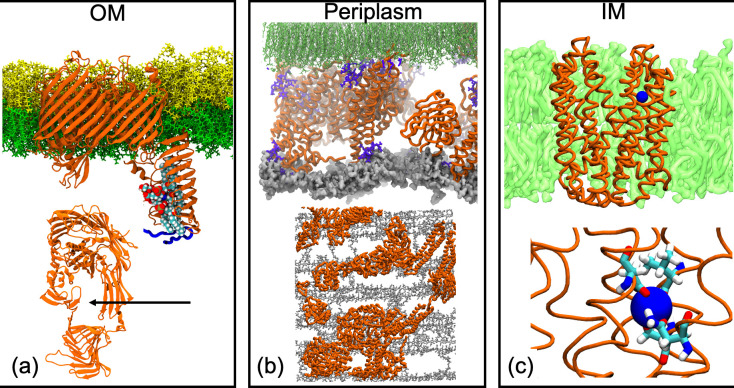
(a) Model of LptDE (orange) with bound antimicrobial peptide (blue) from *K. pneumoniae *, in a model OM (LPS is yellow and phospholipids are green) with LPS molecules in the periplasmic domain of LptD [57]. A rotated view showing the open lateral gate is shown at the bottom. (b) A crowded periplasm model showing cell wall (grey), proteins (orange), the antibiotic polymyxin B1 (purple) and inner leaflet lipid tails of the OM (green) [100]. A rotated (bird’s eye) view from the outer membrane side, in which just the cell wall and proteins are present is shown at the bottom. (c) The ClbM MATE transporter from *E. coli* (orange) in a model inner membrane (green), with a Na^+^ ion (blue) bound in the N-lobe of the protein [75]. A close-up view of the Na^+^ ion coordinated to residues to D53, N195, V213 and S217 is shown in the bottom.

Another area in which our understanding of the cell envelope has benefited significantly from MD simulations is the mechanism of LPS insertion into the outer membrane. LPS is extracted from the inner membrane and inserted into the outer membrane by the cell-envelope-spanning LPS transport (Lpt) translocon. The translocon is comprised of seven proteins, LptA–G. While we discuss simulations of cell-envelope-spanning proteins elsewhere in this review, the Lpt system is described here given that, of the seven proteins, LptD/E are the only ones with multiple reported MD simulations. The integral OMP LptD and the OM-anchored lipoprotein LptE form a heterodimer, which transports LPS from the periplasm to the outer leaflet of the OM. The first structure of the LptD/E complex revealed LptD from *Salmonella Typhimurium* to be a 26-stranded β-barrel and LptE to form a roll-like structure with three-quarters of the protein located inside the β-barrel of LptD. MD simulations identified the weakest part of the barrel, and predicted opening of the LptD channel, allowing passage of LPS by separation of specific beta strands [56]. A further study in which MD simulations were employed with hydrogen deuterium exchange mass spectrometry (HDXMS) showed how binding of LPS to a specific domain of LptD (the beta taco domain) of *Klebsiella pneumoniae* opens up that domain as well as simultaneously inducing conformational rearrangements of the β-strands adjacent to the putative lateral gate (Fig. 2). The authors further showed that the antimicrobial peptide thanatin stabilizes the beta taco domain and acts as a non-competitive inhibitor of LPS transportation [57]. MD simulations initiated from the x-ray structures of *Shigella flexneri* LptD/E complex, as part of a wider collaborative study, highlighted the role of two key proline residues in permitting the passage of LPS by disrupting full beta strand formation and hydrogen-bonding interactions within the residues surrounding the putative lateral gate [58]. More recently, free energy calculations have predicted that the presence of LPS reduces the energetic barrier to opening of the putative lateral gate of LptD by ~10 kcal/mol [59]. In summary, simulations have helped identify the location of the lateral gate of LptD, provided insights into the mechanism and energetics of gate opening, and characterized conformational rearrangements of LptDE in response to LPS binding as well as how these rearrangements are impacted by the presence of antibiotics.

## Inner-membrane proteins

Over the years there have been many reported simulation studies of bacterial inner-membrane proteins and by virtue of the simpler phospholipid composition of the IM (compared to the OM), the simulation systems have always been fairly representative. Some of the earliest IM protein studies were those involving potassium channels, examples include work on KcsA [60, 61] and the inwardly rectifying (KirBac) channels [62, 63]. Indeed, in recent years these channels are still the focus of many experimental and simulation studies given the origins of ion selectivity, mechanisms of the various types of gating and regulation of ion flow are still not completely understood [64, 65]. A number of excellent reviews are devoted to this topic, and we therefore refer the reader to these, simply noting here that ion channel studies do make up a significant portion of the bacterial IM protein simulation literature [66, 67]. Here we discuss simulation contributions to understanding of transport of folded proteins via the twin-arginine protein transport (Tat pathway), extrusion of xenobiotics via the Multidrug And Toxic Compound Extrusion (MATE) transporters and transport of ammonium into the cell via Amt transporters; these examples of IM protein systems have been chosen as multiple simulation studies of each have been reported, but we note here that these are just a few examples of the many IM systems to which simulation studies have made key contributions.

### Tat system

Transport of folded proteins across the cell membranes of prokaryotes occurs via the twin-arginine protein transport (Tat pathway) [68]. Perhaps the most widely studied bacterial Tat system is that of *E. coli,* which is assembled from multiple copies of the membrane proteins TatA, TatB and TatC. The precise details of how the multiple Tat components within the assembled translocation complex are arranged are still elusive, which in part hampers determination of the full mechanism of Tat transport. MD simulations have played a significant role over the years in gaining insights into the assembly and mechanism of the Tat system found in *E. coli* and other bacterial species. Earlier work included the following: (i) simulations that provided support for a ‘charge zipper’ mechanism of TatA translocation [69]; (ii) simulations based on a solution NMR model of the TatA complex of *E. coli,* which suggested that distortion of the local lipid bilayer leading to transient membrane rupture may play a role in the process of protein translocation [70]; and (iii) two studies that include simulations of TatC from the thermophile *Aquifex aeolicus *, both of which provided details of the membrane positioning and orientation of the complex [71, 72].

In an area of intense research with many open questions and areas of controversy, perhaps the most detailed contributions from molecular modelling simulations are those reported by Alcock *et al*. in 2016 [73]. This study combined sequence co-evolution analysis, molecular modelling and simulations, and experimental microbiology to determine the interactions between the proteins of the *E. coli* Tat system. Contact predictions from the co-evolution analysis – which predicted that TatA and TatB bind to the same site on TatC – along with available structural data from *A. aeolicus* were used to build models of the TatAC and TatBC heterodimers. MD simulations of the resultant dimers in phospholipid bilayers demonstrated the stability of both models and provided further support for the plausibility of either TatA or TatB binding to the contact site predicted by co-evolution analysis. These data combined with those from additional simulations in which the protonation states of key residues and mutations of polar residues were explored have provided the most comprehensive molecular simulation-based contribution to date of the interactions and therefore insights into the assembly of the *E. coli* Tat protein translocase. We refer the reader to a recent review for further details of current understanding of the Tat transport [68].

### MATE transporters

The extrusion of xenobiotics is achieved in bacteria via six families of transporters, ATP-binding cassette (ABC), resistance nodulation cell division (RND), major facilitator superfamily (MFS), small multidrug resistance (SMR), proteobacterial antimicrobial compound efflux (PACE) and multidrug and toxic compound extrusion (MATE). Here we discuss simulation contributions to our understanding of the MATE transporters. MATE is a large family of secondary active transporters that in bacteria is involved in the transport of largely cationic compounds, including antibiotics [74]. MATE transporters extrude cationic compounds using either a Na^+^, H^+^ or combined Na^+^/H^+^ gradient for electroneutral exchange. In order to achieve expulsion of their substrates, the transporters must undergo a conformational change from the inward-facing conformation via which the substrate can enter from the cytoplasmic side of the membrane to the outward-facing conformation, which allows the substrate to be extruded into the periplasm. Until 2019, all of the available structures of NorM MATE transporters showed them in the outward-facing state, thus there has been a clear role for MD in providing insights into the full transport cycle. Consequently, a number of simulation studies have explored various MATE transporters including ClbM from *E. coli*, NorM from *N. gonorrhoeae* and *V. cholerae* and PfMATE from *P. furiosus* [75–80]. A key aspect of understanding the mechanism or transport is identification of the cation- and proton-binding sites. Simulations of ClbM suggested two distinct Na^+^ binding sites, one being a site which had been interpreted as being occupied by a water molecule in the original x-ray structure of this protein [75]. Interestingly the simulations predicted that water would not be stably bound within this site (Fig. 2). Na^+^-binding sites have also been predicted from MD simulations of the NorM from *N. gonorrhoeae* and *V. cholerae* and the PfMATE transporters [76, 77, 79]. Additional questions in the transport pathway include the following: (i) Are the Na^+^ binding sites occupied when the substrate is bound or when the protein is in the apo state? (ii) What triggers the conformational rearrangement, Na^+^ binding or drug binding? (iii) What is the nature of the inward facing conformation? A significant development came in 2019, with the crystal structure of PfMATE in the inward-facing state [80]. Targeted MD simulations predicted the transition between the two conformational states of this protein to proceed via rigid body motions of the transmembrane helices. Furthermore, these MD simulations also revealed the ability of anionic lipids to enter the central cavity of PfMATE when the protein is in the outward-facing conformation. The simulations described here highlight how MD simulations can yield detailed insights into the mechanism of xenobiotic transport, which may facilitate the battle against antibiotic resistance. We discuss further examples of simulations of biomedically relevant efflux pumps, namely RND and ABC transporters, in Cell-envelope-spanning protein complexes in the context of envelope-spanning protein complexes.

### Amt

In Gram-negative bacteria, ammonium exchange is mediated by the ammonium transporter/methylammonium-ammonium permease (Amt/Mep) proteins. While Mep proteins have been the focus of MD studies, there has been greater focus on the AmtB, ammonium transporter from *E. coli* [81]. The molecular mechanisms of ammonium transport are not fully characterized given that the available structures of AmtB show the transporter in the inward-facing state regardless of the presence/absence of ammonium, and thus unsurprisingly the AmtB transporter has been the focus of a number of MD simulation studies. Simulations in combination with electrophysiology and yeast functional complementation experiments have shown that ammonium is transported across the membrane via a ‘two-lane’ mechanism, and specifically, H^+^ and NH_3_ are transported separately following NH_4_^+^ deprotonation [82]. Interestingly, a study in which MD simulations and pKa calculations were reported predicted that the ammonium ion only becomes deprotonated when it enters the interior of the channel [83]. A number of putative ammonium binding sites have been identified including Am1 (the first binding site from the periplasmic end of the protein). The greater importance of the periplasmic entrance of the protein over the cytoplasmic exit in terms of selectivity was identified from a series of equilibrium MD simulations [84]. A subsequent simulation study predicted that the periplasmic vestibule selectively recruits solutes with the following preference: NH_4_^+^ > NH_3_> CO_2_ to the Am1 site [85]. This selectivity was thought to occur via flipping of two phenylalanine residues. The same study also showed the role of an aspartate residue, which had been shown to be essential for activity, predicting it to be important for the structural integrity of the periplasmic vestibule of the protein.

Another intriguing aspect of Amt structure and function is related to the local membrane environment. Mass spectrometry and structural biology studies showed that AmtB binds the anionic lipid POPG specifically, which stabilizes the conformation of the protein [86]. A combination of MD simulation and *in vitro* studies subsequently revealed additional POPG binding sites near the subunit interfacial regions [87]. It was shown that without POPG binding, AmtB is not functional as an ammonium transporter. The simulations did not reveal any large topological changes in the protein upon POPG binding. The most notable observation was the preferred formation of a short helix within a periplasmic loop region in the presence of POPG and while this may have functional relevance, currently the precise role played by the lipid is still unclear. Thus, MD simulations have provided insights into how the Amt structure is stabilized, how it selects solutes, and how the structure is impacted by the lipids of the membrane.

## Periplasmic proteins

The periplasm is the aqueous compartment that divides the two membranes and houses the cell wall as well as many soluble proteins, some of which may bind to the cell wall and/or be anchored in one of the membranes. The periplasm also contains a plethora of non-protein macromolecules such as those collectively referred to as osmolytes.

Periplasmic binding proteins (PBPs) undergo large conformational changes upon binding ligands. A number of PBPs have been studied by MD simulations. One example is the maltose/maltodextrin-binding protein (MBP) of the maltose transport system. Structures of the protein in the apo and ligand-bound forms are known, and these two states correspond to open and closed conformations, respectively. Atomistic MD simulations have revealed the transitions between the open and closed (and partially closed) conformations of the protein upon addition and removal of ligands, with identification of key interactions that stabilize each conformation [88, 89]. Similar comparative conformational dynamics have been reported for BtuF, a component of the vitamin B12 uptake system of *E. coli*, which showed greater conformational lability in the apo form compared to the ligand bound form of the protein [90]. Similar results were also reported for the ribose-binding protein, also from *E. coli* [91]. Here, while the structures of the proteins in their different states were known, the simulations were able to predict the pathways that link the states, thereby providing mechanistic insights.

The only protein known to be covalently bound to the cell wall in *E. coli* is also the most abundant protein in the bacterium; Braun’s lipoprotein (Lpp). In agreement with experimental studies, MD simulations have shown the impact of the length and conformation of Lpp on the width of the periplasm [16, 92]. Insights into proteins that form non-covalent interactions with the cell wall such as OmpA, Pal and TolR have also come from MD simulations [92–96]. The first reported MD simulation study to incorporate models of both membranes and the cell wall of *E. coli* revealed a flat cell wall when proteins formed non-covalent interactions from the both OM and IM sides, whereas local undulations were observed when proteins bound from only one side [92]. This level of atomistic detail of the cell wall as a function of protein binding is very difficult to achieve with current experimental techniques. The lipoprotein carriers LolA and LolB have been the focus of a number of simulation studies. LolA carries the lipoproteins across the cell wall and delivers them to LolB, which is anchored to the OM via a lipid moiety at its N-terminus. Simulations have shown that the hydrophobic cavities of both proteins are fairly indiscriminate. Spontaneous binding of small hydrophobic inhibitor molecules was observed from atomistic MD simulations of LolA [97]. This study predicted that such inhibitor molecules work by reducing the affinity of LolA for their lipoprotein substrates. Stansfeld and co-workers, as part of a larger study of lipoproteins, showed that the LolB-lipoprotein interaction is energetically more favourable than LolA-lipoprotein, thereby likely revealing the driving force for transfer of lipoproteins from LolA to LolB [98].

Two studies have sought to characterize macromolecular dynamics within crowded models of the periplasm. A study by Lalgudi and Elcock incorporated 13 of the most abundant *E. coli* periplasmic proteins and the cell wall [99]. While the resulting simulations were limited to tens of microseconds, the study was important in establishing computational protocols for constructing such systems. One of the most complex models of the periplasm reported to date is the study of periplasmic crowding by Pedebos *et al*. [100] (Fig. 2). A number of models were investigated, all including the OM containing OmpA, the cell wall and Lpp linking the cell wall and the OM. The models differed in the number of periplasmic proteins and presence/absence of osmolytes and the antibiotic polymyxin B1. It was shown that greater crowding leads to reduction in the diffusion rates of water, proteins and other macromolecules. A range of intermolecular interactions were identified and interestingly it was noted that polymyxin B1, which is a lipoprotein itself, may bind into the hydrophobic cavities of LolA and LolB, suggesting a possible route for antibiotic passage through the periplasm. Interactions of the proteins, osmolytes and antibiotics with the cell wall were also characterized.

## Cell-envelope-spanning protein complexes

Here we discuss progress in understanding mechanistic aspects of two cell-spanning transport systems in which simulations have played a significant role; the RND efflux pumps and the maintenance of lipid asymmetry (Mla) phospholipid transport system.

### RND efflux pumps

An innate antibiotic resistance mechanism in Gram-negative bacteria originates from their ability to expel antibiotics from their cytoplasm via cell-envelope-spanning efflux pumps. The mechanisms via which antibiotics are recognized and expelled by these pumps have been the foci of much research effort in recent years, with MD simulations also playing their part [101, 102]. Perhaps the most widely studied efflux pump is the AcrAB-TolC pump from *E. coli*, with simulations reported of individual components, as well as more recently, the entire cell-envelope-spanning complex [16]. For the sake of simplicity, we will focus largely on this pump here. The AcrAB-TolC efflux pump consists of AcrB in the inner membrane, TolC in the OM, and the fusion protein AcrA, which connects the inner- and outer-membrane-spanning proteins. A number of insights into the conformational behaviour of this pump alone (i.e. without drugs) have come from MD simulations. Examples include, but are not by any means restricted to the following: (i) the conformational dynamics of TolC, particularly at its periplasmic mouth from a number of studies [103–105]; (ii) allosteric effects due to protonation states of AcrB; and more recently [106] (iii) prediction of conformations of AcrA corresponding to pump assembly-competent and assembly-incompetent states of the protein [107]. The complete expulsion of antibiotics across the envelope is not straightforward to characterize with computational methods given that the IM process is energy dependent. However, significant progress has been made in understanding aspects of the individual components of the overall process of drug expulsion. For example, a recent systematic study of the interaction of eight carbapenem antibiotics with the distal pocket (DP) of AcrB predicted how the physicochemical characteristics of the antibiotics may impact their binding preference [108]. In particular, Atzori *et al*. found a correlation between the molecular weight of the carbapenems and the specific location of sub-pockets within the DP to which they preferentially bind. This could represent a general feature of the polyspecificity of AcrB compared to other investigated properties, such as total charge or fraction of hydrophobic and polar molecular surface areas, which could not be correlated to the binding preference of the carbapenems. Additionally, the simulation-generated data from this work are particularly significant given direct efflux measurements for the carbapenems are not available from experimental studies.

A number of key MD studies of efflux pumps from other Gram-negative bacteria have also been reported. For example, Ruggerone and co-workers predicted the location of binding sites within MexB, an AcrB homologue from *P. aeruginosa,* for the antibiotics meropenem and imipinem, as well as their relative binding affinities [109]. The x-ray structure of the OqxB RND pump from *K. pneumoniae *, which showed electron density corresponding to n-dodecyl-β-d-maltoside (DDM) molecules in the substrate-binding pocket, was augmented by docking and MD simulations from which the binding mode of the fluoroquinolones ciprofloxacin, levofloxacin and moxifloxacin was predicted [110]. Simulations have also contributed to the understanding of the mechanism of action of inhibition of efflux pumps although these are not reviewed here.

### The Mla system

The Mla proteins are a cell-envelope-spanning complex responsible for the transport of phospholipids between the cell interior and the OM. While structures of all the system components are now known, the direction of lipid transport is still debated. Studies of the Mla system in *Acinetobacter baumannii* provide support for both retrograde (OM to IM) and anterograde (IM to OM) transport of phospholipids. It is not our intention here to give an opinion/support for either model of transport, but rather to present the details of what has been learnt about this system from MD simulations. The complex is comprised of MlaA present in the OM, MlaC in the periplasmic space and the MlaBDEF ABC transporter system in the IM; simulations of all of these components have been reported. Prior to the determination of the structure of MlaA, a model was used in a study exploring MlaA interaction with the trimeric porin OmpC [111]. MD simulations showed the structural fold of the predicted MlaA model to be more stable in a lipid bilayer than in an aqueous environment. A combination of cross-linking data and rigid-body MD were employed to predict a model of the OmpC-MlaA complex, which was then subjected to unrestrained dynamics. The results revealed that MlaA is located inside the membrane, tucked into the dimeric interface of the OmpC trimer. Kleinekathöfer and co-workers reported simulations of MlaA, which were performed as part of a collaborative study with experimental groups [112]. The high-resolution structure of MlaA was reported for the first time as part of this study. MD simulations provided support for the protein being embedded in the inner leaflet of the OM and predicted that it does not interact with LPS in the outer leaflet. MlaA was shown to be a monomeric α-helical OMP forming a ring-like topology with a central, amphipathic pore, with MD simulations showing that water molecules rapidly fill the central MlaA channel, and thus that it can accommodate polar molecules. During the simulations, PE phospholipids remained stably bound near the MlaA channel constriction in the centre of the bilayer and thus it was postulated that a slight conformational rearrangement would be needed to widen the channel for passage of the lipids towards the periplasm.

**Fig. 3.**
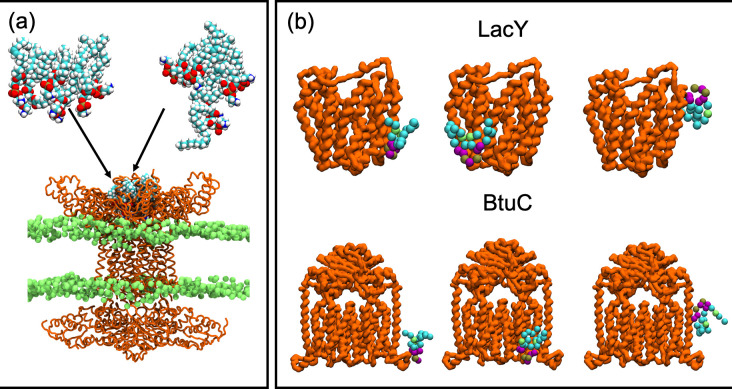
(a) The MlaBDEF complex (orange) from *A. baumannii* in a model inner membrane (green) [113]. The lipids in the MlaD basket region at the start of the simulation are shown in the top left along with their locations after 250 ns of simulation, top right. (b) Representative cardiolipin-binding sites predicted from CG simulations for LacY (top) and BtuC (bottom) [124].

Simulations of the IM component, the MlaBDEF complex of the Mla system of *A. baumanni* were reported in a study in which cryo-EM structures of this complex were also published [113]. Interestingly, in addition to the proteins, density corresponding to detergent molecules was also seen in two distinct locations; in the ‘basket’ region of MlaD which is exposed to the periplasm, and within pockets formed by transmembrane helices of MlaD and MlaE. MD simulations showed that phospholipid molecules can bind stably within both regions. Indeed, lipids were observed to enter the MlaD/MlaE pockets even during the equilibration stage of the simulations. Seven phospholipids were modelled into the MlaD basket with their lipid tails facing the periplasm and their headgroups facing the cytoplasm and arranged with one lipid in the centre of the ring formed by the other six lipids. Intriguingly, in one simulation, the central lipid was observed to re-orientate such that its lipid tails were facing toward the cytoplasm, and while the other six lipids remained stably bound in their original positions, the re-orientated lipid was observed to move further towards the cytoplasm (Fig. 3). Thus, molecular simulations of the Mla OM components have provided insights into MlaA location within the IM and its ability to accommodate polar molecules as well as details of its interactions with porins, and simulations of the IM components have helped to rationalize structural data by providing support for phospholipid binding sites within the complex.

## Lipid–protein interactions

Thus far we have focussed on specific proteins, but it is increasingly clear that membrane protein function is greatly impacted and often modulated by interactions with the lipids within the local membrane, and in turn the membrane properties are affected by the native proteins (as we have mentioned above in particular with respect to LPS-OMP interactions) [114].

Shearer *et al*. showed that the complex asymmetrical mixture of lipids present in *E. coli* OMs have unique patterns of interaction with six different membrane proteins of the *E. coli* OM, OmpA, OmpX, BtuB, FhuA, OmpF and EstA, pointing to a role for LPS in positioning and orientating the proteins in the membrane [115]. An approach integrating MD with mass spectrometry showed that PG lipids interact specifically with OmpF and maintain the porin in the open state [116]. A similar study in which mass spectrometry and simulations were employed together showed that protein–lipid interactions can be sensitive to the saturation level of phospholipid tails [117].

Cardiolipin is an important functional lipid in the IMs of Gram-negative bacteria, and has been shown experimentally to interact with many bacterial IM proteins, for example AmtB, MurJ, LeuT and AqpZ [86, 118–120]. Simulations allow for the identification and characterization of cardiolipin interaction sites, which sheds light on the functional role of specific cardiolipin sites. For example, cardiolipin is required for the proper function of SecY, via direct protein–lipid interactions and the transient nature of the interactions indicate a possible role in proton translocation [121]. The aforementioned AcrB, which is a component of the RND efflux pump of *E. coli*, forms a complex with a small protein AcrZ; the latter was shown via simulations to lead to enrichment of cardiolipin in the local vicinity of the protein complex [122]. In a similar manner, cardiolipin enrichment around the protein was observed from simulations of MurM, an enzyme from the Gram-positive bacterium *S. aureus* and experimental functional assays were shown to enhance enzyme activity when the cardiolipin content of the bilayer was increased [123]. Simulation of the Uracil/H+symporter, UraA, from *E. coli* revealed three cardiolipin-binding sites on the surface of the protein, two in the inner leaflet and one in the outer leaflet. It was hypothesized that cardiolipin may play a role in moving protons to and from UraA within cardiolipin enriched areas of the *E. coli* IM.

While such details of individual cardiolipin–protein interactions are undoubtedly important, characterization of general patterns of behaviour call for comparative studies. To this end, an extensive simulation study of lipid interaction with all 42 *E. coli* IM proteins of known structure was reported by Corey *et al*. [124]. The study showed that cardiolipin interacts particularly on the inner leaflet side of the protein, and identified that cardiolipin sites typically comprise two to three basic residues which should be in the same membrane plane, one or more polar residues, and an aromatic residue towards the membrane core (Fig. 3).

## Outlook

In summary, in developing our current understanding of the molecular organization and physicochemical characteristics of the cell envelope of Gram-negative bacteria – including mechanistic and environment-dependent behaviour of specific cell-envelope proteins, MD simulations have played a significant and unique role. Simulation has augmented experimental data, helped to interpret otherwise puzzling and sometimes contradictory data from different experimental sources, and increasingly is generating new hypotheses. The simulation field has matured significantly over several decades, particularly in the context of increasingly realistic membrane models, and we reiterate that there are now far more high quality and biologically insightful bacterial membrane simulation studies than we can explicitly mention here. Thus, we have focused primarily on instructive recent examples, which we believe demonstrate the advancements in MD models and methods that have enabled simulation of membranes with lipidic compositions, which more closely resemble their *in vivo* counterparts, periplasmic models with realistic levels of crowding, and cell envelope models incorporating all three compartments.

Current limitations in the scope of MD as a tool for studying bacterial cell envelopes can be divided into general groups: those arising from a lack of structural or experimental detail for the system of interest, and those where the computational models and methodologies require further development. For example, in the former case, the precise details of the structure and morphology of the cell wall for most bacterial species is unavailable. Likewise, an example of the latter case is the ongoing difficulty in quantifying kinetics from simulations of crowded systems. Nevertheless, with increasing numbers of researchers working in the field, perhaps in part motivated by the looming threat posed by antimicrobial resistance to antibiotics, progress is being made in all of these areas. The advent of high-throughput structural methods and the ‘resolution revolution’ in cryo-EM, which is yielding ever larger structures at near-atomic resolution along with advances in cryo-tomography have already made a huge impact as discussed above. Developments in the computational toolkit are keeping up with structural biology via, for example continued improvement of enhanced sampling methods, and the development of models, which range from all-atom resolution to the mesoscale, the latter allowing for simulation of system sizes that are often comparable to their experimental counterpart. This is further strengthened by the enormous emerging potential of machine learning and related methodologies. Thus, with continued collaborative efforts between computational and experimental researchers and sufficient support for high-performance computing, the coming years will doubtless yield exciting new insights into the structure, dynamics and functioning of bacterial cell envelopes.
